# Nitrogen stress response and stringent response are coupled in *Escherichia coli*

**DOI:** 10.1038/ncomms5115

**Published:** 2014-06-20

**Authors:** Daniel R. Brown, Geraint Barton, Zhensheng Pan, Martin Buck, Sivaramesh Wigneshweraraj

**Affiliations:** 1 MRC Centre for Molecular Bacteriology and Infection, Imperial College London, London SW7 2AZ, UK; 2 Centre for Systems Biology and Bioinformatics, Division of Biosciences, Imperial College London, London SW7 2AZ, UK; 3 Department of Life Sciences, Imperial College London, London SW7 2AZ, UK

## Abstract

Assimilation of nitrogen is an essential process in bacteria. The nitrogen regulation stress response is an adaptive mechanism used by nitrogen-starved *Escherichia coli* to scavenge for alternative nitrogen sources and requires the global transcriptional regulator NtrC. In addition, nitrogen-starved *E. coli* cells synthesize a signal molecule, guanosine tetraphosphate (ppGpp), which serves as an effector molecule of many processes including transcription to initiate global physiological changes, collectively termed the stringent response. The regulatory mechanisms leading to elevated ppGpp levels during nutritional stresses remain elusive. Here, we show that transcription of *relA*, a key gene responsible for the synthesis of ppGpp, is activated by NtrC during nitrogen starvation. The results reveal that NtrC couples these two major bacterial stress responses to manage conditions of nitrogen limitation, and provide novel mechanistic insights into how a specific nutritional stress leads to elevating ppGpp levels in bacteria.

## Abstract (web summary)

The nitrogen regulation stress response and the stringent response are two major regulatory pathways in bacteria, but their mutual coordination is unclear. Here, the authors show that NtrC, the main regulatory protein from the first pathway, activates expression of *relA*, a key gene from the second pathway.

## Graphical abstract

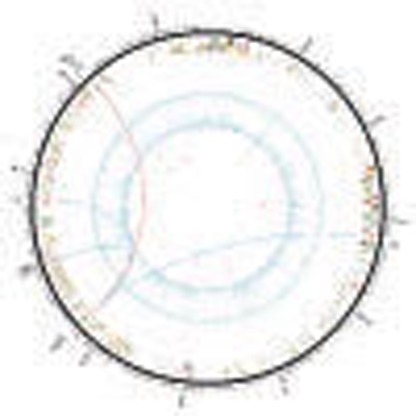

Nitrogen (N) is an essential element of most macromolecules in a bacterial cell, including proteins, nucleic acids and cell wall components. Bacteria can assimilate a variety of N sources and ammonia typically supports the fastest growth, serving as the preferred N source for many bacteria including *E. coli*^1^. During N-limited growth ammonia is converted by glutamine synthetase into glutamine, the primary amine donor for key amino acid and nucleotide biosynthetic pathways. *E. coli* cells respond to N starvation by activating the nitrogen regulation (Ntr) stress response, resulting in the expression of ~100 genes to facilitate N scavenging from alternative sources of combined N (ref. 2). In enterobacteria, the master transcription regulator of the Ntr stress response is NtrC of the NtrBC two-component system^3^. N limitation results in the phosphorylation of the response regulator NtrC (product of *glnG*) by its cognate sensor kinase NtrB (product of *glnL*).

N-starved *E. coli* cells rapidly synthesize ppGpp, the effector alarmone of the bacterial stringent response^4^. Two enzymes modulate the levels of ppGpp in *E. coli*: the ppGpp synthetase RelA and ppGpp synthetase/hydrolase SpoT^5^. RelA and SpoT contribute to stress adaptation, antibiotic tolerance, expression of virulence traits and the acquisition of persistent phenotype in pathogenic bacteria^6^,^7^,^8^,^9^. The regulation of RelA/SpoT enzyme activities has been extensively studied^10^,^11^,^12^,^13^, but the regulatory mechanisms that govern transcription of their genes and thereby lead to elevated ppGpp levels during nutritional stresses remain elusive.

Here, we address the links between the Ntr stress response and the ppGpp alarmone. To gain insight into NtrC-dependent gene networks, we map the genome-wide binding targets of NtrC and RNA polymerase (RNAp) during N starvation in *E. coli*, using chromatin immunoprecipitation followed by high-throughput sequencing (ChIP-seq). Our results reveal that nitrogen stress response and stringent response are coupled in *E. coli*.

## Results

### Tooling up to study genome-wide binding of NtrC and RNAp

**Figure 1:**
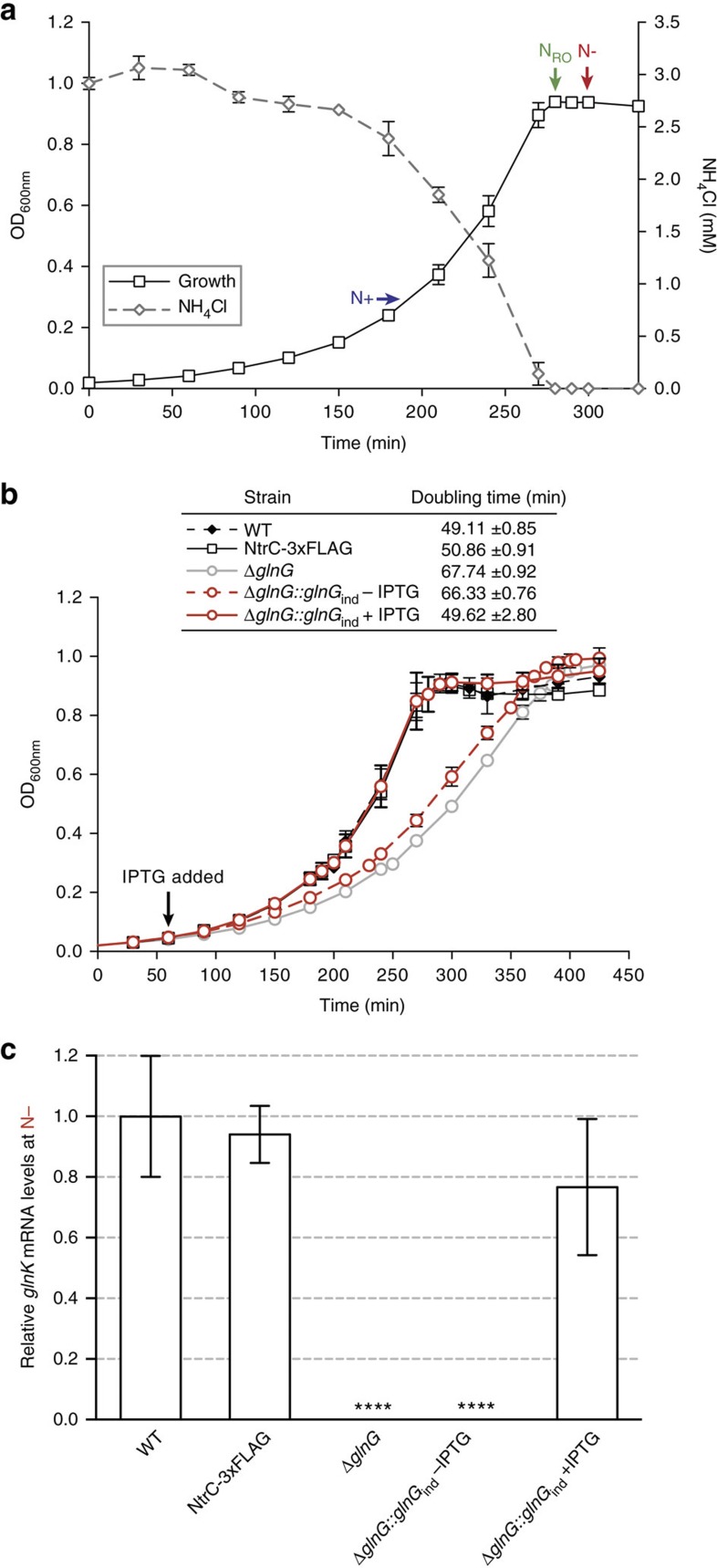
Establishing N-starved growth conditions in *E. coli*. (**a**) The growth arrest of wild-type *E. coli* NCM3722 cells coincides with ammonium run out (at *t*=N_RO_) in the minimal Gutnick medium. The time points at which the *E. coli* cells were sampled for downstream analysis are indicated (*t*=N+ and *t*=N− represents growth under nitrogen replete and starved conditions, respectively). (**b**) The growth curves of wild-type NCM3722, NCM3722:*glnG*-FLAG (NtrC-3xFLAG), NCM3722:Δ*glnG* and NCM3722:Δ*glnG::glnG*_*ind*_ (−/+ IPTG). The quantitation of the doubling times is also given. (**c**) Graph showing the relative levels of *glnK* mRNA expression as fold-change in cells sampled at *t*=N+ and *t*=N−. Error bars on all growth curves represent s.d. (where *n*=3). Statistical significant relationships from One-way ANOVA analysis are denoted (****P<0.0001).

To identify genome regions preferentially associated with NtrC, we introduced an in-frame fusion encoding three repeats of the FLAG epitope at the 3′ end of *glnG* in *E. coli* strain NCM3722, a prototrophic *E. coli* K-12 strain. To establish N-starved conditions, we grew batch cultures of the wild-type NCM3722 strain in Gutnick minimal medium supplemented with a limiting amount (3 mM) of NH_4_Cl as the sole N source and monitored bacterial growth as a function of ammonium consumption. In Fig. 1a, the time when wild-type NCM3722 stops growing (*t*=N_RO_) coincides with the ammonium run out ([ammonium]<0.000625, mM) in the growth medium. To establish that the FLAG epitope had not compromised the activity of NtrC, we measured the doubling times of wild-type NCM3722, NCM3722:*glnG*-FLAG and NCM3722:Δ*glnG* strains under our growth conditions. The doubling time of the NCM3722:*glnG*-FLAG strain is close to that of the wild-type NCM3722 strain; however, as expected, the doubling time of the NCM3722:Δ*glnG* strain is longer than that of the wild-type NCM3722 strain (Fig. 1b). Further, the expression levels of *glnK* mRNA, an Ntr stress response gene directly activated by NtrC, at *t*=N− (that is, the time-point 20 min after N runs out) are similar in the wild-type NCM3722 and NCM3722:*glnG*-FLAG strains, and as expected is not readily detected in the NCM3722:Δ*glnG* strain (Fig. 1c). Complementation of the NCM3722:Δ*glnG* strain with inducible plasmid-borne *glnG* restored both the doubling time and *glnK* mRNA expression (Fig. 1b,c). In summary, we conclude that (i) FLAG-tag has not adversely affected biological activity of NtrC and (ii) under our experimental conditions *t*=N− is representative of the time when the *E. coli* cells are starved for N.

### NtrC binds upstream of *relA* in N-starved *E. coli*

**Figure 2:**
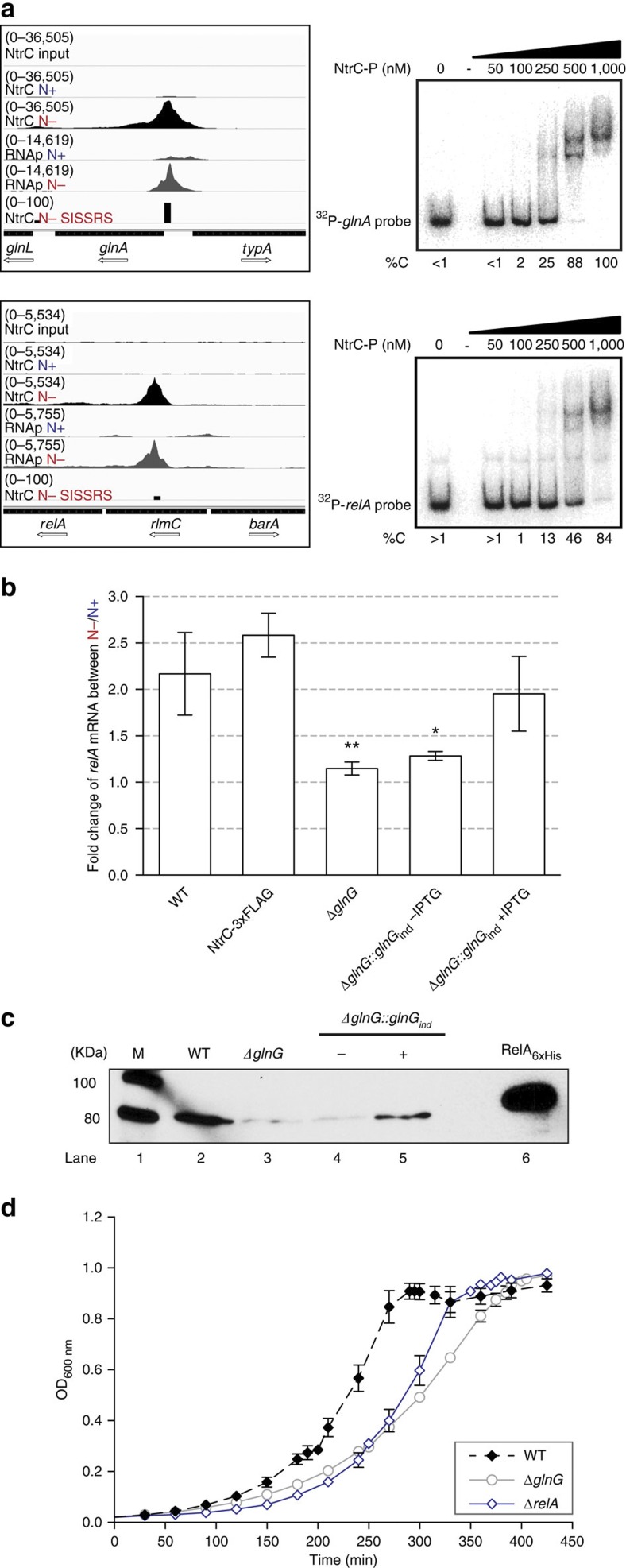
NtrC binds to a site upstream of *relA* in N-starved *E. coli*. (**a**) *Left*. Shown are screenshots of Integrative Genome Viewer with tracks showing the binding profiles (tag density) as measured by ChIP-seq of NtrC (black) and RNAp (grey) in N-non-starved (denoted as N+) and N-starved (denoted as N−) *E. coli* aligned against the upstream regions of *glnA* and *relA*. Tracks with the input DNA control tag density (denoted as input) and with the genomic loci bound by NtrC identified by the peak-calling algorithm Site Identification for Short Sequence Reads (denoted as SISSRS) at *t*=N− are also shown for comparison. *Right*. Representative autoradiographs of non-denaturing gels showing the binding of NtrC to ^32^P-labelled DNA probes with sequences corresponding to the upstream regions of *glnA* (positions −273 to +71 relative to the translation-start site of GlnA) and *relA* (positions −928 to −592 relative to the translation-start site of RelA). The %C indicates the percentage of DNA bound by NtrC in comparison with unbound DNA in lane 1. (**b**) Graph showing the relative levels of *relA* mRNA expression as fold-change in cells sampled at *t*=N+ and *t*=N−. The error bars represent s.d. and statistical significant relationships from one-way ANOVA analysis are denoted (*P<0.05; **P<0.01). (**c**) Representative autoradiograph of a western blot (full gel image in Supplementary Fig. 8) showing expression of RelA proteins in cells sampled at *t*=N−. Lane 1 contains the molecular weight marker and lane 6 contains purified *E. coli* RelA-6xHis protein. (**d**) The growth curves of wild-type NCM3722, NCM3722:Δ*glnG* and NCM3722:Δ*relA*.

To identify the genomic loci bound by NtrC in *E. coli* during N starvation, we sampled cells for ChIP-seq analysis at *t*=N− and NtrC-bound DNA was precipitated with anti-FLAG antibodies. To identify transcriptionally active promoters in *E. coli* during N starvation, we treated the *E. coli* cells at *t*=N− with rifampicin for 15 min (rifampicin inhibits transcription elongation and thus transcription-initiated RNAp molecules will become trapped at any functional promoter^14^,^15^) and RNAp bound DNA was precipitated with a monoclonal antibody against the β-subunit of the RNAp. The ChIP-seq data revealed that NtrC bound upstream of at least 21 transcription units at *t*=N−. RNAp binding (in the presence and absence of Rif treatment) to promoter regions of all 21 transcription units and differential gene expression analysis (*t*=N− versus *t*=N+) indicate that all 21 transcription units are upregulated at *t*=N− (Table 1, Supplementary Figs 1–3). Fourteen of the 21 transcription units are known members of the NtrC regulon (Table 1)^2^. However, none of the seven remaining transcription units (*flgMN, dicC, fliC, relA, ssrS, soxR* and *yjcZ-proP*) have been previously demonstrated to require NtrC for their transcriptional regulation. Therefore, we tested NtrC binding to these regions by electrophoretic mobility shift assays (EMSA) with purified *E. coli* NtrC and DNA probe sequences (designed following ChIP-seq analysis) upstream of *flgMN, dicC, fliC, relA, ssrS, soxR* and *yjcZ-proP* (referred to as *flgN*, *dicC*, *fliC*, *relA*, *ssrS*, *soxR* and *yjcZ* probes). As a positive control, the region upstream of the *glnALG* the operon (referred to as the *glnA* probe) was used. The *in situ* phosphorylated NtrC (NtrC-P) bound to the *relA* and positive control *glnA* probes (Fig. 2a) but not the other probes (Supplementary Fig. 2). ChIP-seq data indicate that the NtrC binding site is located between positions −838 and −649 upstream from the translational start of RelA. Since complex formation between NtrC-P and *flgN*, *dicC*, *fliC*, *ssrS*, *soxR* and *yjcZ* probes is not detectable even at a higher concentration of NtrC-P (Supplementary Fig. 2) and the DNA regions are poorly enriched by NtrC binding in the ChIP-seq data (Table 1), we consider that *flgN*, *dicC*, *fliC*, *ssrS*, *soxR* and *yjcZ* are unlikely to be solely dependent on NtrC. We do not exclude the possibility that the upstream regions of *flgN*, *dicC*, *fliC*, *ssrS*, *soxR* and *yjcZ* transcription units contain intrinsically poor NtrC binding sites, which function in co-dependence with other transcription factors (Supplementary Fig. 2).

### NtrC activates *relA* transcription in N-starved *E. coli*

To unambiguously establish that *relA* transcription is activated by NtrC during N starvation, we measured *relA* mRNA levels in wild-type NCM3722 and NCM3722:Δ*glnG* strains at *t*=N− relative to *t*=N+. In the wild-type NCM3722 strain, the level of *relA* mRNA expression is ~2.2±0.4-fold at *t*=N− relative to *t*=N+, and no detectable increase in the *relA* mRNA expression is seen in the NCM3722:Δ*glnG* at *t*=N− relative to *t*=N+ (Fig. 2b). The complementation of the NCM3722:Δ*glnG* strain with inducible plasmid-borne *glnG* restored *relA* mRNA expression in the NCM3722:Δ*glnG:glnG*_*ind*_ strain to levels comparable to that of the wild-type NCM3722 strain in the presence of the inducer isopropyl β-D-1-thiogalactopyranoside (IPTG) (Fig. 2b). To determine whether synthesis of the RelA protein correlates with NtrC-dependent activation of *relA* transcription, we probed whole-cell extracts of the wild-type NCM3722, NCM3722:Δ*glnG* and NCM3722:Δ*glnG::glnG*_*ind*_ strains sampled at *t*=N− with a monoclonal antibody to *E. coli* RelA. As expected, RelA protein was not detected in whole-cell extract prepared from the NCM3722:Δ*glnG* strain and NCM3722:Δ*glnG::glnG*_*ind*_ strain in the absence of IPTG (Fig. 2c, lanes 3 and 4); however, RelA protein was detected in whole-cell extracts prepared from the NCM3722:Δ*glnG::glnG*_*ind*_ strain in the presence of IPTG (Fig. 2c, lane 5). These results support our ChIP-seq data and demonstrate that accumulation of RelA in *E. coli* is NtrC dependent during N starvation. Further the growth of NCM3722:Δ*glnG* and NCM3722:Δ*relA* strains under N-limiting conditions is similar (Fig. 2d), thus indicating that NtrC-dependent activation of RelA is important for the tolerance of N stress.

### A novel σ^54^-dependent promoter drives transcription of *relA*

NtrC exclusively activates transcription from promoters used by RNAp containing the major variant bacterial σ factor, σ^54^ (ref. 16). At such promoters, hexamers of phosphorylated NtrC bind to enhancer-like sequences located ~100–150 nucleotides upstream of the transcription start site and in a reaction hydrolysing adenosine triphosphate (ATP), convert a transcriptionally inactive initial σ^54^-RNAp-promoter complex to a transcriptionally active one^17^. A previous study revealed that two promoters, P1 and P2, which are utilized by the housekeeping σ^70^-RNAp, drive the transcription of *relA*^18^. The P1 promoter is constitutively active, but, on entry into stationary phase, *relA* transcription is driven by P2 and it is thought that transcription from P2 is influenced by the global transcriptional regulator cAMP receptor protein (CRP). To identify σ^54^-RNAp-dependent promoter(s) responsible for driving NtrC-dependent transcription of *relA*, we carried out 5′-RACE analysis of *relA* mRNA isolated from N-starved wild-type NCM3722 and NCM3722:Δ*glnG E. coli* cells and mapped the transcription start sites (TSS). Results shown in Supplementary Fig. 4A indicate that (i) in N-starved *E. coli*, *relA* is transcribed from three different promoters, the constitutive σ^70^-RNAp-dependent P1 promoter and two new promoters, which we refer to as P3 and P4 and (ii) transcription from P3 and P4 is dependent on NtrC. Transcription from the σ^70^-RNAp dependent P2 promoter was only detected in the NCM3722:Δ*glnG* cells, indicating that σ^54^-RNAp binding to the P4 promoter (which is located immediately adjacent to the P2 promoter and overlaps the CRP binding site; Supplementary Fig. 4B) is likely to antagonize efficient transcription initiation from P2 under N starvation (Supplementary Fig. 4A). Inspection of the DNA region immediately upstream of the TSS of the P3 and P4 transcripts revealed two conserved sequences typical of a σ^54^-RNAp-dependent promoter^19^ (Supplementary Fig. 4B). Since the ChIP-seq data indicate that the DNA region from −811 to −607 (with respect to the translation start site of RelA) is enriched for RNAp binding and spans the P4 promoter (Fig. 2a and Supplementary Fig. 4B), we suggest that NtrC preferentially activates transcription from the P4 promoter in N-starved *E. coli* rather than from the P3 promoter. To demonstrate *in vitro* that P4 represents a *bona fide* NtrC and σ^54^-RNAp-dependent promoter, we conducted a small primed RNA (spRNA) synthesis assay^20^ using purified *E. coli* NtrC, σ^54^ and RNAp and using purified *E. coli* NtrC, σ^54^ and RNAp and a promoter DNA fragment (encompassing sequences −261 to +75 with respect to P4 TSS) and thus containing the NtrC binding site and P4 promoter region. Since the sequence of the template strand at P4 is GACC (position −1 to +3), we used the spRNA assay to monitor the accumulation of the ^32^P labelled and dinucleotide CpU-primed RNA product (CpUpGpG) in the presence α^32^P-GTP. The CpUpGpG RNA product is only synthesized under conditions when NtrC is phosphorylated *in situ* with carbamyl phosphate and σ^54^-RNAp is present (Supplementary Fig. 4C, lanes 4 and 5). Notably, an alignment of the *relA* regulatory sequences from several representatives of the Enterobacteriaceae family indicates that the regulatory mechanism involving NtrC and the σ^54^-RNAp that governs the transcription of *relA* under N starvation is conserved (Supplementary Fig. 5).

### NtrC couples the Ntr and stringent responses in *E. coli*

**Figure 3:**
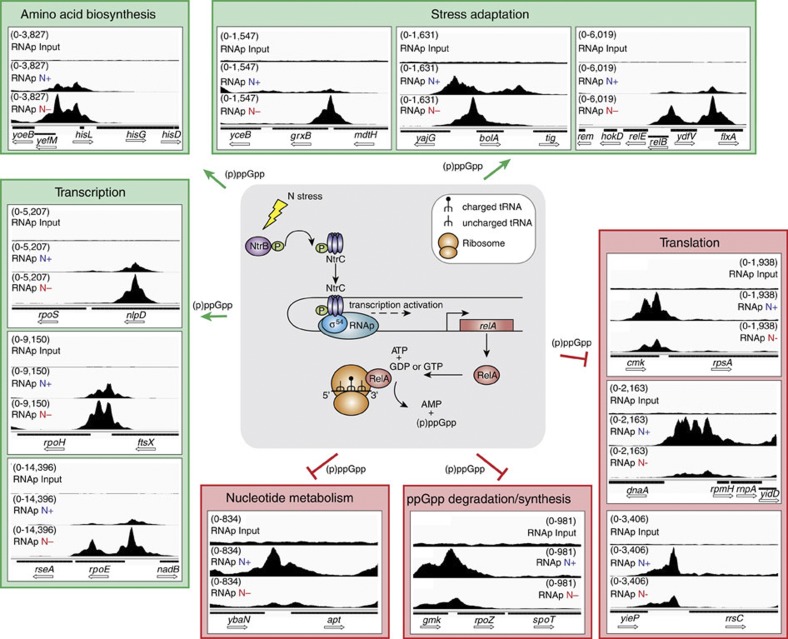
NtrC couples the Ntr stress response and stringent response in N-starved *E. coli.* The cartoon in the middle of the figure represents the model by which N starvation is sensed and leads to the NtrC-mediated activation of transcription of *relA*, which subsequently leads to the production of ppGpp. Around this cartoon, we show screenshots of Integrative Genome Viewer^32^ with tracks showing the binding profiles (tag density) as measured by ChIP-seq of RNAp binding in N-non-starved (denoted as N+) and N-starved (denoted as N−) *E. coli* aligned against the upstream regions of a representative set of known ppGpp responsive promoters grouped into key cellular processes. A track with the input DNA control tag density (denoted as input) is shown for comparison. The screenshots in the green and red boxes denote promoters at which RNAp binding is positively and negatively, respectively, affected by ppGpp.

Zimmer *et al.*^2^ previously concluded that the Ntr stress response represents a scavenging response since many of the NtrC-activated genes encode transport systems of N-containing compounds. The new results provided in this study reveal that during N starvation *E. coli* uses NtrC to integrate the need to scavenge for N with stringent-response-mediated changes that enable cells to adapt to low N availability. By directly activating the expression of RelA and so elevating the intracellular concentration of ppGpp, the effector molecule of stringent response, cells are expected to further reprogramme gene expression. In support of this view, a small increase in *relA* expression has been shown to profoundly increase intracellular ppGpp levels^12^. Since transcription initiation is a major regulatory target of ppGpp, which binds to the RNAp and either positively or negatively affects its ability to form transcriptionally proficient complexes with the promoter^8^,^9^,^21^,^22^, we examined the binding profiles of RNAp under N+ and N− growth conditions at 60 ppGpp responsive promoters listed on the EcoCyc database^23^. The results reveal the expected RNAp binding profiles at 35 of the 60 ppGpp responsive promoters, thereby clearly demonstrating that NtrC stimulates the stringent response in N-starved *E. coli* (Fig. 3 and Supplementary Fig. 6). RNAp binding is positively affected at promoters of genes involved in transcriptional reprogramming (for example, *rpoS*, *rpoH* and *rpoE*), amino-acid biosynthesis (for example, *hisLGDCBHAFI*) and stress adaptation (for example, *bolA, relEB* and *grxB*). RNAp binding is negatively affected at promoters of genes involved in translation (for example, *rpsA, rpmH, rnpA, rrsC*), nucleotide metabolism (for example, *apt*) and ppGpp degradation (*spoT*). However, RNAp binding at 22 of the 60 ppGpp responsive promoters is unaffected in N-starved *E. coli* and 3 display altered binding that is not expected (Supplementary Fig. 6). Results from differential gene expression analysis (*t*=N− versus *t*=N+) are consistent with the ChIP-seq data to a large extent and reveal that 37 out of 60 promoters display the expected transcriptional activity at *t*=N− (Supplementary Table 1). In *E. coli* and several other bacteria, ppGpp is also the major regulator of bacterial persistence, a phenomenon characterized by slow growth and tolerance to antibiotics and other environmental stresses; a family of genes known as toxin-antitoxin (TA) pairs play a pivotal role in the acquisition of the persistence phenotype^6^. We note that RNAp binds to promoters of 9 of the 12 TA genes found in *E. coli* under N− but not N+ conditions, thus implying that N starvation could cause a shift towards a persistent phenotype (Supplementary Fig. 7).

## Discussion

The principal finding of this study is that NtrC, the master regulator of the Ntr stress response, activates the transcription of *relA* from a σ^54^-dependent promoter in N-starved *E. coli* and other members of the Enterobacteriaceae family. A physiological theme for σ^54^-dependent gene regulation has yet to emerge, as the σ^54^-dependent genes described to date control a diverse set of cellular processes^24^,^25^. The finding that transcription of *relA* in N-starved *E. coli* exclusively depends on the RNAp containing the σ^54^ promoter specificity factor further underscores the fundamental importance of σ^54^ to bacterial physiology. Previous studies demonstrated that the Ntr stress response is amplified through the transcriptional activation of the global transcription factor nitrogen assimilation control protein (Nac) by NtrC^2^,^26^. Our results reveal a far wider ranging control of genes occurs through a direct interface of the master Ntr stress response transcription activator NtrC with the bacterial stringent response system. The results predict that NtrC-mediated activation of RelA expression will lead to elevated intracellular levels of ppGpp, which, although consistent with multiple independent observations from gene expression and ChIP-seq data, requires to be directly confirmed in future studies. Nevertheless, the discovery that the regulation of RelA expression, which synthesizes one of the major effectors of stringent response, ppGpp, is integrated into the Ntr stress response in N-starved *E. coli* and possibly in other enteric bacteria provides a paradigmatic example of the dynamic complexity of bacterial transcription regulatory networks. Such behaviour allows bacteria to efficiently adapt to nutritional adversity and demonstrates how bacteria may acquire new metabolic states in response to stress.

## Methods

### Bacterial strains, plasmids and growth conditions

All strains used in this study were derived from *E. coli* NCM3722, a derivative of *E. coli* K-12 strain MG1655. The NCM3722:Δ*glnG* strain was constructed as described previously^27^; briefly, the knockout *glnG* allele was transduced using the P1vir bacteriophage with JW3839 from the Keio collection^28^ serving as the donor strain and NCM3722 as the recipient strain. The kanamycin cassette was then cured from the strain using pCP20 leaving an unmarked knockout mutation. The NCM3722:Δ*relA* strain was constructed in the same way with *E. coli* JW2755 (Keio collection) serving as the donor strain^28^. The NCM3722:*glnG*-FLAG strain (NtrC-3xFLAG) was constructed by introducing an in-frame fusion encoding three repeats of the FLAG epitope (3 × (gat tac aag gat gac gat gac aag)), to the 3′ end of *glnG*. A *glnG*-inducible complementing plasmid (pAPT-glnG-rbs31) was kindly provided by Wang *et al.*^29^ Bacteria were grown in Gutnick media (33.8 mM KH_2_PO_4_, 77.5 mM K_2_HPO_4_, 5.74 mM K_2_SO_4_, 0.41 mM MgSO_4_), supplemented with Ho-LE trace elements^30^ and 0.4% glucose, and containing either 10 mM NH_4_Cl (for precultures) or 3 mM NH_4_Cl (runout experiments) as the sole nitrogen source at 37 °C, 200 r.p.m. Ammonium concentrations in the media were determined using the Aquaquant ammonium quantification kit (Merck Millipore, UK), according to the manufacturer’s instructions.

### Antibodies

For immunoblotting, commercial mouse monoclonal anti-RelA (5G8) (at a diution of 1/200) was used (Santa Cruz, USA) in conjunction with the anti-mouse ECL horseradish peroxidase (HRP)-linked secondary antibody (at a dilution of 1/10,000) (GE Healthcare, UK). For chromatin immunoprecipitation (ChIP), mouse monoclonal anti-Flag (M2, Sigma-Aldrich, UK), 10 μl per individual immunoprecipitation experiment, and mouse monoclonal antibody to β-subunit of *E. coli* RNAp (WP002 Neoclone, USA), 4 μl per individual immunprecipitation experiment, were used.

### ChIP-seq

Cells were grown as stated above in Gutnick minimal media, when growth reached the correct stage (for N+, when OD_600nm_=0.3, and for N−, 20 min after growth arrest due to nitrogen runout) samples were taken and cells were subjected *in vivo* crosslinking with the addition of formaldehyde (Sigma, UK) (final concentration 1% (v/v)). For RNAp-rif ChIP only, cells were subjected to a 15-min treatment with rifampicin (final concentration 150 μg ml^−1^) at 37 °C before crosslinking. Crosslinking was carried out for 20 min at 37 °C before it was quenched by the addition of glycine (final concentration 450 mM) for 5 min at 37 °C. Cells were harvested from 20 ml of culture by centrifugation, washed twice in Tris-buffered saline (pH 7.5) and pellets were frozen at −80 °C until required. To fragment the cellular DNA, thawed pellets were resuspended in immunoprecipitation (IP) buffer (50 mM HEPES-KOH pH 7.5, 150 mM NaCl, 1 mM EDTA, 1% (v/v) Triton X-100, 0.1% (w/v) Na deoxycholate, 0.1% (w/v) SDS) supplemented with complete protease inhibitor (Roche, UK) before sonication (100% amplitude, 30 s pulses for 10 min) (Misonix Ultrasonic Processor S-4000), this resulted in fragments of 200–400 bp. Cell debris was removed by centrifugation and the supernatant recovered for IP. A 100-μl aliquot of the supernatant was removed and stored at −20 °C to act as the ‘input’ sample, which would serve as the background control for ChIP-seq, this sample was de-crosslinked and subjected to protein degradation as described for the ‘test’ samples.

Either anti-FLAG (M2) or anti-β (WP002) was added to the remaining supernatant (~750 μl) and incubated overnight at 4 °C on a rotating wheel (a no-antibody control IP was set up at this point also). Sheep anti-Mouse IgG Dynal beads (Invitrogen, UK) were prepared by washing 2 × with PBS and 2 × with IP buffer, before saturating the beads (IP buffer supplemented with complete protease inhibitor and 1 mg ml^−1^ BSA) overnight at 4 °C on a rotating wheel. The blocking solution was removed by applying the beads to a magnet (which was the method used throughout the IP to harvest bead complexes) and removing the supernatant, antibody-nucleoprotein complex was added and incubated for 2 h at 4 °C on a rotating wheel. Bead-antibody-nucleoprotein complexes were harvested and subjected to a series of washing steps; 2 × IP buffer, 1 × IP salt buffer (IP buffer+0.5 M NaCl), 1 × Wash buffer III (10 mM Tris pH 8, 250 mM LiCl, 1 mM EDTA, 0.5% Nonidet-P40, 0.5% (w/v) Na deoxycholate) and a final wash in TE buffer (50 mM Tris, 10 mM EDTA pH 7.5). Immunoprecipitated complexes were then eluted from the beads using an elution buffer (50 mM Tris–HCl pH 7.5, 10 mM EDTA, 1% (w/v) SDS) and incubation at 65 °C for 40 min with regular agitation. Beads were removed using the magnet and the eluate carefully removed and diluted twofold in nuclease-free water (VWR, UK). DNA was purified from the eluate by de-crosslinking and degrading the protein by incubation at 42 °C for 2 h and 65 °C for 6 h in the presence of Pronase (final concentration 4 mg ml^−1^) before Qiagen MiniElute Kit purification (this was carried out for the ‘input’ sample described above also). DNA was quantified using the high sensitivity dsDNA Qubit assay (Invitrogen, UK).

Libraries of ChIP-purified DNA were prepared to allow multiplex next generation sequencing using Illumina TruSeq DNA sample preparation kit v2 (Illumina, USA) according to the manufacturer’s instructions with the following modifications. Ten nanograms of ChIP-purified DNA was used to construct each library, barcoded adapters were diluted 10-fold to compensate for the lower DNA amount. An additional 5 cycle PCR was added before size selection of libraries to improve yields (PCR completed as described in amplification of libraries in the kit with PCR primers provided), also an additional gel extraction step was added following final PCR amplification to remove excess primer dimers. PCR amplification for NtrC ChIP libraries was completed using KAPA HiFi HotStart Readymix (Kapa Biosystems, USA) instead of the kit polymerase as this improved amplification of libraries. Size and purity of DNA was confirmed using high sensitivity DNA analysis kit on an Agilent 2100 Bioanalyser (Agilent, USA). DNA libraries were multiplexed and sequenced using an Illumina HiSeq2000, which generated ~10 million reads per sample. Reads were mapped to the complete genome of *E. coli* strain MG1655 (NCBI reference sequence: NC_000913.2) using Bowtie^31^. Reads were visualized and screenshots prepared using Integrative Genome Viewer (IGV)^32^. All ChIP-seq data files have been deposited into ArrayExpress (accession code E-MTAB-2211). Genomic loci bound by NtrC and RNAp were identified using the peak-calling algorithm SISSRs (Site Identification for Short Sequence Reads)^33^, with peaks defined as significant with a *P*-value of < 0.005 and if they showed greater than 9-fold enrichment in the immunoprecipitated sample compared with the input control.

### Quantitative real-time PCR (qRT-PCR)

Total RNA samples were extracted from cells at specified time points, RNA was stabilized with Qiagen RNA protect reagent (Qiagen, USA) and extracted using the PureLink RNA Mini kit (Ambion Life Technologies, USA). Purified RNA was stored at −80 °C in nuclease-free water. cDNA was amplified from 100 ng of RNA using the high-capacity cDNA reverse transcription kit (Applied Biosystems, USA). qRT-PCR reactions were completed in a final volume of 10 μl (1 μl cDNA, 5 μl TaqMan Fast PCR master mix, 0.5 μl TaqMan probe (both Applied Biosystems, USA)). Amplification was performed on an Applied Biosystems 7500 Fast Real-Time machine using the following conditions; 50 °C 5 min, 95 °C 10 min, and 40 cycles of 95 °C 15 s, 60 °C 1 min. Real-time analysis was performed in triplicate on RNA from three independent cultures and quantification of 16S expression served as an internal control. The relative expression ratios were calculated using the delta-delta method (PerkinElmer, USA). Statistical analysis of data was performed by one-way ANOVA, a *P*-value ≤0.01 was considered to be a significant difference. Primer and probe mixtures were custom designed from Applied Biosystems (Custom TaqMan gene expression Assays (Applied Biosystems, Life Technologies, USA)) sequences can be viewed in Supplementary Table 2.

### Microarray analysis of gene expression

Total RNA from *E. coli* cells sampled at *t*=N− and *t*=N+ were extracted as above, and samples were processed by OGT (Oxford, UK) to incorporate Cy3 or Cy5 dyes and hybridized onto OGT 4 × 44 K high-density oligonucleotide arrays. Gene expression data were collected by scanning the array using an Agilent Technologies microarray scanner, and results were extracted by using Agilent Technologies image-analysis software with the local background correction option selected. Statistical analyses of the gene expression data was carried out using the statistical analysis software environment R together with packages available as part of the BioConductor project ( http://www.bioconductor.org). Data generated from the Agilent Feature Extraction software for each sample was imported into R. Replicate probes were mean summarized and quantile normalized using the preprocess Core R package. The limma R package^34^ was used to compute empirical Bayes moderated *t*-statistics to identify differentially expressed gene between time points. Generated *P*-values were corrected for multiple testing using the Benjamini and Hochberg false discovery rate (FDR). An FDR-corrected *P*-value cutoff of <0.01 was used to determine significant differential expression. Differential gene expression results only for the 60 ppGpp responsive genes are provided in Supplementary Table 1.

### Proteins

Core RNA polymerase subunits α2ββ′ω (collectively known as E), σ^54^ and PspF_1-275_ were expressed and purified as described in detail previously^35^. In brief, core RNA polymerase was purified by nickel-affinity chromatography through the 6 × -His-tagged β-subunit following previous ammonium sulphate precipitation and heparin column purification of cell lysate. σ^54^ was purified by successive precipitation steps using streptomycin sulphate (2% final concentration) and ammonium sulphate (70% final concentration) followed by ion-exchange chromatography using a Sepharose column. Finally, the sample was subjected to heparin column purification to separate σ^54^ from the core RNA polymerase. PspF_1-275_ was expressed as an N-terminally 6 × -His-tagged protein from a pET28b+ based plasmid and purified by nickel-affinity chromatography^35^. 6 × -His-tagged NtrC from ASKA(-) JW3839 (ref. 36) was purified by nickel-affinity chromatography as follows. In brief, cells were grown at 37 °C in 80 ml LB medium supplemented with 30 μg ml^−1^ chloramphenicol to an OD_600nm_ 0.3. Cultures were induced with 0.4 mM IPTG (final concentration) for a further 2 h before samples were taken. The cells were collected by centrifugation at 4,500 *g* for 30 min, and the pellet was stored at −20 °C. Thawed pellets were resuspended in Ni buffer A (25 mM NaH_2_PO_4_, pH 7.0, 500 mM NaCl, and 5% (v/v) glycerol) containing a cocktail tablet of protease inhibitors (Complete, Roche Diagnostics, USA) and lysed using probe sonication. Soluble protein extract was recovered following centrifugation at 20,000 *g*, this was loaded onto a pre-charged nickel column (GE Healthcare, UK) and purified via affinity chromatography using a AKTA prime FPLC (GE Healthcare, UK) as described for σ^54^ and PspF_1-275_ (ref. 35). Pooled fractions containing 6 × -His-tagged NtrC were dialysed against storage buffer (10 mM Tris–HCl, pH 8.0, 50 mM NaCl, 0.1 mM EDTA, 1 mM DTT, and 50% (v/v) glycerol) at 4 °C overnight. Aliquots were then stored at −80 °C until required.

### Small primed RNA synthesis assay

Small primed RNA (spRNA) synthesis assays were conducted as previously described^20^. In brief, assays were performed in STA buffer (25 mM Tris-acetate, pH 8.0, 8 mM Mg-acetate, 10 mM KCl and 3.5% w/v PEG 6000) in a 10-μl reaction volume containing 50 nM Eσ^54^ (reconstituted using a 1:4 ratio of E:σ^54^) and 20 nM promoter DNA probe (*relA* probe sequence used as template DNA can be seen in Supplementary Table 3), which was initially incubated for 5 min at 37 °C to form inactive Eσ^54^-DNA complexes. To this, NtrC (+/− phosphorylation with Carbamoyl phosphate) (final concentration 1 or 2 μM) or PspF_1-275_ (final concentration 1 μM) was added, for open complex formation dATP was added at 4 mM (final concentration), samples were incubated for a further 5 min at 37 °C. Synthesis of spRNA (CpUpGpG) was initiated by the addition of a mix containing 0.5 mM CpU, 4 μCi [α-32P]GTP, 0.05 mM GTP and incubated for 60 min at 37 °C. The reaction was stopped by addition of 4 μl loading dye (80% formamide, 10 mM EDTA, 0.04% bromophenol blue, and 0.04% xylene cyanol) and 3 min incubation at 95 °C. Products were analysed by PAGE on denaturing 20% TBE-urea gels, run at 200 V for 40 min, and visualized and quantified using a Typhoon FLA 7000 PhosphorImager (GE Healthcare, UK). All experiments were performed at least in triplicate.

### Electrophoretic mobility shift assay

Electrophoretic mobility shift assays (EMSA) were conducted in Reaction buffer (40 mM Tris–HCl (pH 8.0), 100 mM NaCl, 10 mM MgCl2, 1 mM DTT) in a total-reaction volume of 10 μl containing 0, 50, 100, 250, 500 or 1,000 nM NtrC and 10 mM Carbamyl phosphate, incubated for 30 min at 37 °C before the addition of 5 nM ^32^P-labelled probe (sequences can be found in Supplementary Table 3), following which the reaction was incubated for a further 10 min at 37 °C. Reactions were analysed on 4.5% (w/v) native polyacrylamide gel. The gel was run for 60 min at 100 V at 37 °C and then dried. NtrC-DNA probe complexes were visualized and quantified using a Typhoon FLA 7000 PhosphorImager (GE Healthcare, UK).

### Transcription start site mapping by 5′RACE PCR

Transcription start site mapping was conducted using a 5′ RACE System for Rapid Amplification of cDNA Ends, Version 2.0 Kit (Invitrogen, Life Technologies, USA) as described in the manufacturer’s guidelines. For this, RNA was isolated from cells under nitrogen starvation (N−) as described above, sequences for primers used can be found in Supplementary Table 4.

## Additional information

**Accession codes:** Data from the ChIP-seq experiments and from the microarray experiments have been deposited in the ArrayExpress database under accession codes E-MTAB-2211 and E-MTAB-2612, respectively.

**How to cite this article:** Brown, D. R. *et al.* Nitrogen stress response and stringent response are coupled in *Escherichia coli*. *Nat. Commun.* 5:4115 doi: 10.1038/ncomms5115 (2014).

## Supplementary Material

Supplementary Information Supplementary Figures 1-8, Supplementary Tables 1-4 and Supplementary References

## Figures and Tables

**Table 1:** Summary of genome-wide binding sites of NtrC in N-starved *E. coli* determined by ChIP-seq.

**Peak number**	**Genomic loci**	**Fold**	***P*** **-value**	**Gene/Operon**	**Pathway/process**	**Reference**
**1**	471741–471821	39.71	0.0004	*glnK-amtB*	GlnK - Nitrogen regulatory protein, AmtB - ammonia transport	Gene expression analysis^2^
**2**	688381–688461	55.29	0.0003	*gltIJKL*	Glutamate / aspartate ABC transport	Gene expression analysis^2^
**3**	847261–847341	74.78	0.0002	*glnHPQ*	Glutamine ABC transporter	Direct binding^37^, gene expression analysis^2^
**4**	1073221–1073301	94.03	0.0001	*ycdMLKJIHG*	Pyrimidine degradation	Gene expression analysis^2^
**5**	1129181–1129261	13.65	0.0022	*flgMN*	Regulation of flagellar synthesis and flagellar biosynthesis protein	
**6**	1561101–1561181	24.1	0.0009	*ddpXABCDF*	D-ala-D-ala dipeptide transport and dipeptidase	Gene expression analysis^2^
**7**	1646021–1646101	9.49	0.0042	* dicC *	DNA-binding transcriptional repressor	
**8**	1830021–1830101	23.35	0.0009	*astCADBE*	Arginine catabolic pathway	Direct binding^38^, gene expression analysis^2^
**9**	1864821–1864901	12.11	0.0027	*yeaGH*	Unknown (YeaG is a serine protein kinase)	Gene expression analysis^2^
**10**	2001461–2001541	9.11	0.0045	* fliC *	Flagellar biosynthesis component	
**11**	2059941–2060021	59.59	0.0002	*nac-cbl*	Nitrogen limitation response- adapter for σ70 dependent genes	Gene expression analysis^2^
**12**	2424941–2425021	23.73	0.0009	*hisJQMP*	Histidine ABC transporter	Gene expression analysis^2^
**13** *	2425861–2425941	5.38	0.015	* argT *	lysine/arginine/ornithine ABC transporter	Gene expression analysis^2^
**14**	2912341–2912421	13.89	0.0022	* relA *	GDP pyrophosphokinase involved in stringent response	
**15**	3054061–3054141	10.4	0.0036	* ssrS *	6S RNA involved stationary phase regulation of transcription	
**16**	3217421–3217501	10.64	0.0035	* ygjG *	Putrescine degradative pathway	Gene expression analysis^2^
**17**	3416981–3417061	16.21	0.0017	*yhdWXYZ*	Polar amino acid transport	Gene expression analysis^2^
**18**	4056061–4056141	70.98	0.0002	*glnALG*	Glutamine biosynthesis pathway (ammonia assimilation) & nitrogen regulation	Direct binding^39^,^40^ Gene expression analysis^2^
**19**	4054381–4054461	9.42	0.0042	*glnLG*	NtrBC two component system - nitrogen regulation	Direct binding^41^, gene expression analysis^2^
**20**	4275021–4275101	10.22	0.0037	* soxR *	SoxR transcriptional regulator	
**21**	4327301–4327381	11.7	0.0029	*yjcZ-proP*	Hypothetical protein (YjcZ), osmolyte:H+ symporter (ProP)	

^*^Miscalled peak by SISSRs, added from visual interpretation of the ChIP-seq results.
