# Supplementary material for: Nitrogen stress response and stringent response are coupled in Escherichia coli
Source: Nat Commun. 2014 Jun 20;5:4115. doi: 10.1038/ncomms5115 (PMC4066584; doi:10.1038/ncomms5115)
Supplement: Supplementary Information — Supplementary Figures 1-8, Supplementary Tables 1-4 and Supplementary References [file ncomms5115-s1.pdf]

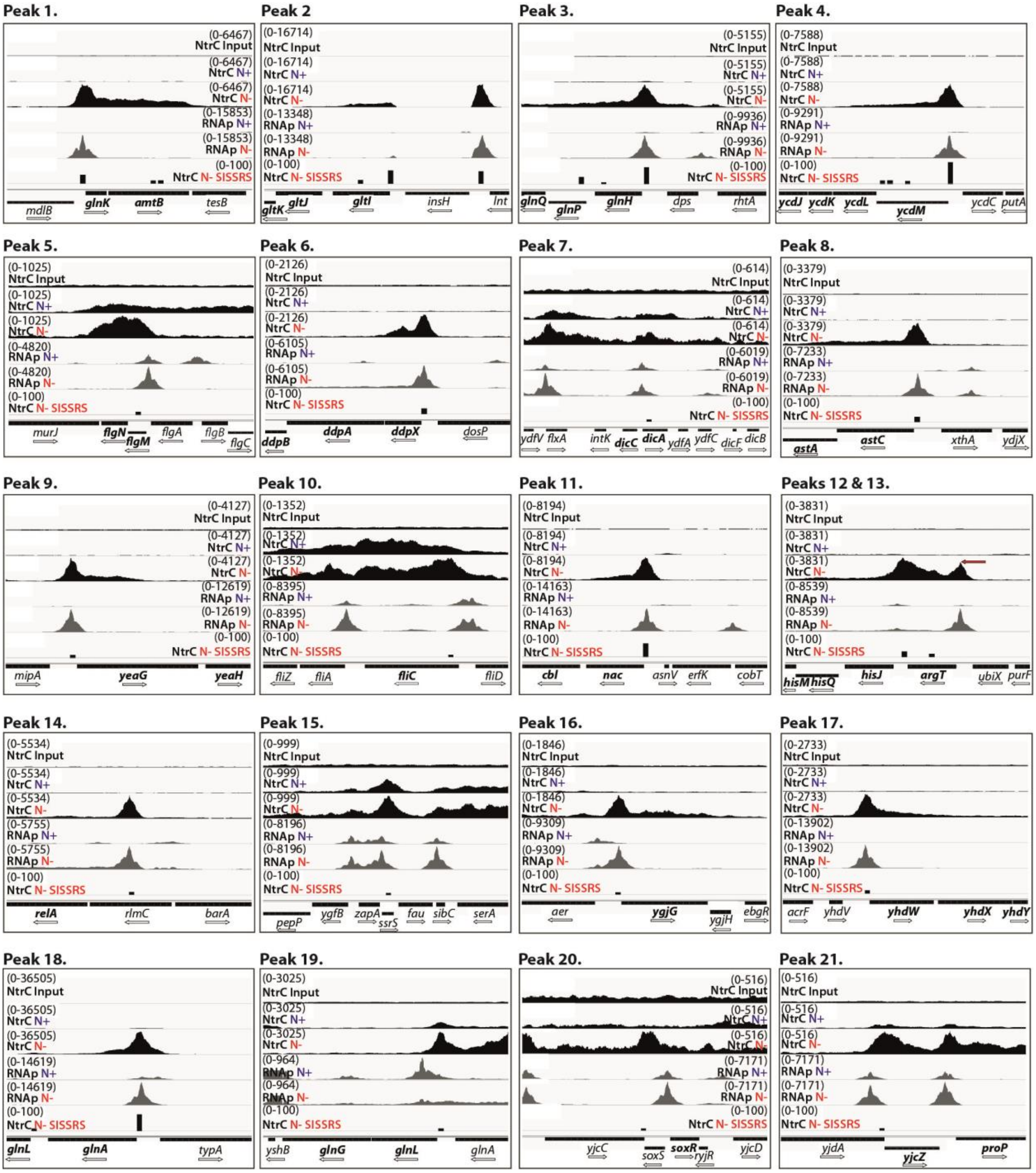

Supplementary Figure 1. Screenshots of Integrative Genome Viewer[1] with tracks showing the binding profiles (tag density) as measured by ChIP-seq of NtrC (black) and RNAp (gray) in N non-starved (denoted as N+) and N starved (denoted as N-) *E. coli* aligned against the upstream regions of all the transcription units shown in Table 1. Tracks with the input DNA control tag density (denoted as input) and with the genomic loci bound by NtrC identified by

the SISSRS peak-calling algorithm [2] at t=N- are also shown for comparison. Red arrow (panel “Peaks 12 & 13”) denotes peak upstream from *argT* gene miss-called by SISSRS.

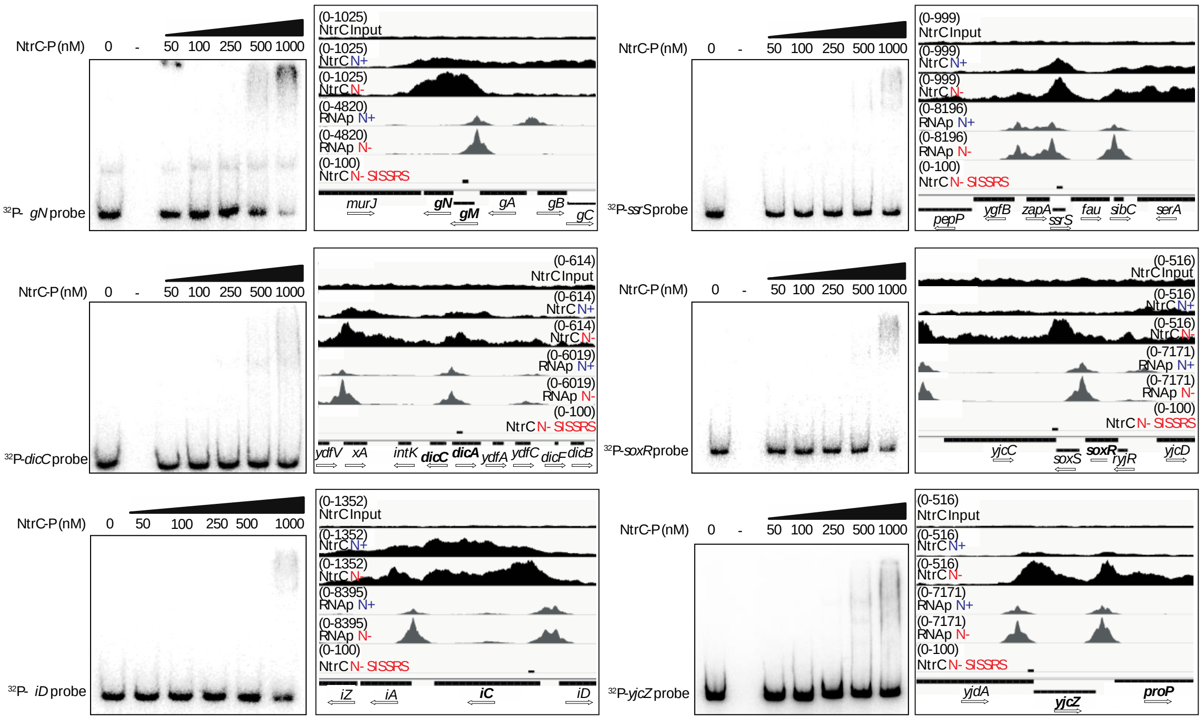

Supplementary Figure 2. Screenshots of Integrative Genome Viewer [1] with tracks showing the binding profiles (tag density) as measured by ChIP-seq of NtrC (black) and RNAp (gray) in N non-starved (denoted as N+) and N starved (denoted as N-) *E. coli* aligned against the upstream regions of transcription units *flgMN*, *dicC*, *fliC*, *ssrS*, *soxR* and *yjcZ-proP*. Here these are supplemented with representative autoradiographs of non-denaturing gels showing the binding of *in situ* phosphorylated NtrC to ^{32}P-labelled DNA probes with sequences corresponding to their respective upstream regions. Tracks with the input DNA control tag density (denoted as input) and with the genomic loci bound by NtrC identified by the SISSRS peak-calling algorithm [2] at t=N- are also shown for comparison.

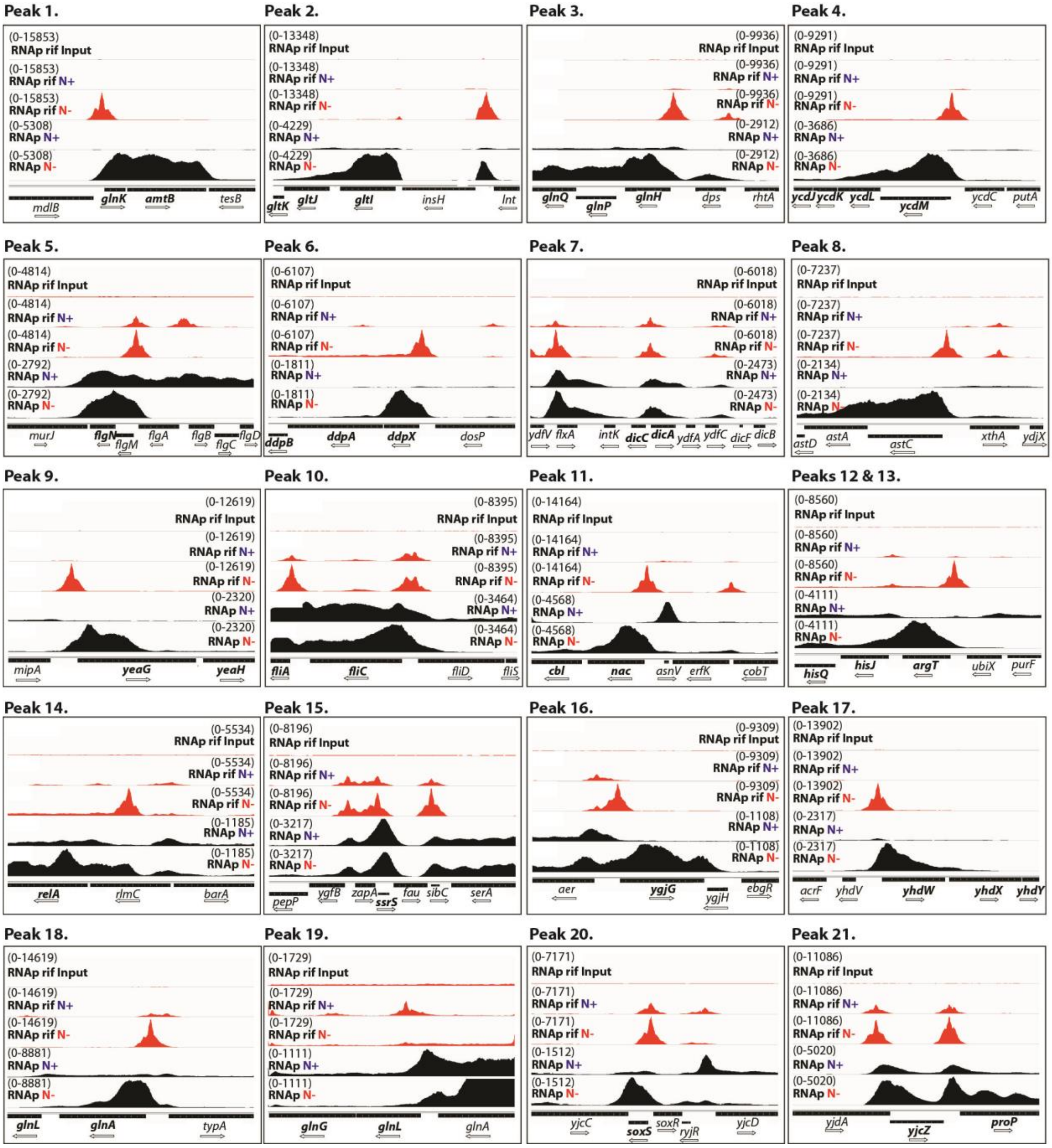

Supplementary Figure 3. Screenshots of Integrative Genome Viewer[1] with tracks showing the binding profiles (tag density) as measured by ChIP-seq of RNAp with Rifampicin treatment (red) and RNAp in the absence of Rifampicin treatment (black) in N non-starved (denoted as N+) and N starved (denoted as N-) *E. coli* aligned against the upstream regions of all the transcription units shown in Table 1. Tracks with the input DNA control tag density (denoted as input) is shown for comparison.

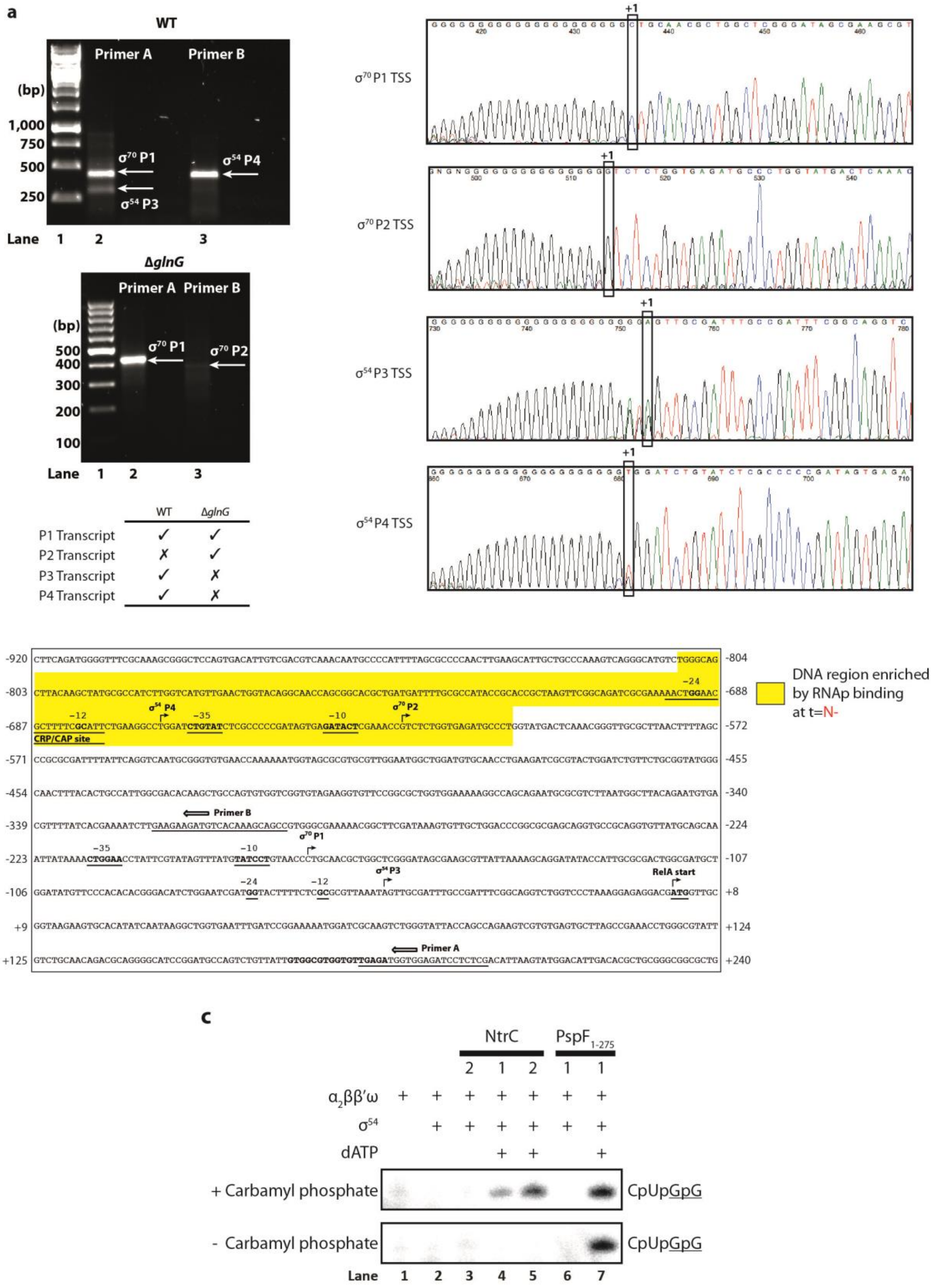

Supplementary Figure 4. Transcription start site mapping and NtrC-activated σ^{54}-dependent transcription of *relA* from novel promoter P4 (**A**) *Left*. Representative image of agarose gels showing the 5'-RACE PCR products amplified from cDNA synthesised from total RNA

isolated from N starved wild-type NCM3722 and NCM3722:Δ*glnG* *E. coli* cells. The presence or absence of the different transcripts originating from promoters P1-4 in the N starved wild-type NCM3722 and NCM3722:Δ*glnG* *E. coli* is shown in the table below the agarose gel images. *Right*. DNA sequencing chromatograms (from FinchTV[3]) showing the transcription start sites (boxed) originating from promoters P1-4 in the regulatory region of *relA* determined by 5'-RACE analysis. (**B**) The DNA sequence of the *relA* regulatory region (from +240 to –920 with respect to the translation start site of RelA). The consensus -10/-35 of P1 and P2 promoters and -12/-24 elements of the P3 and P4 are shown in bold typeface and underlined; the CRP binding site is indicated and underlined. The transcription start sites of P1-P4 and the translation start site of RelA are indicated. The sequences of primers A and B used for making the cDNA for the 5'-RACE analysis are underlined and marked with an empty leftward pointing arrow. The DNA region (from -607 to -811 with respect to the translation start site of RelA) enriched in the immunoprecipitated sample by RNAp binding is highlighted in yellow. (**C**) Representative autoradiographs of 20% (w/v) denaturing urea gel showing the *in situ* phosphorylated NtrC dependent synthesis of CpUpGpG transcript (underlined nucleotides are α^{32}P labelled) from the P4 promoter by σ^{54}-RNAp. The reaction components present in each lane are given at the top of the autoradiographs; lanes 6 and 7 are control lanes and show that the synthesis of CpUpGpG can be initiated from P4 by a non-DNA binding and phosphorylation-independent form of the σ^{54}-RNAp transcription activator $PspF_{1\text{-}275}$.

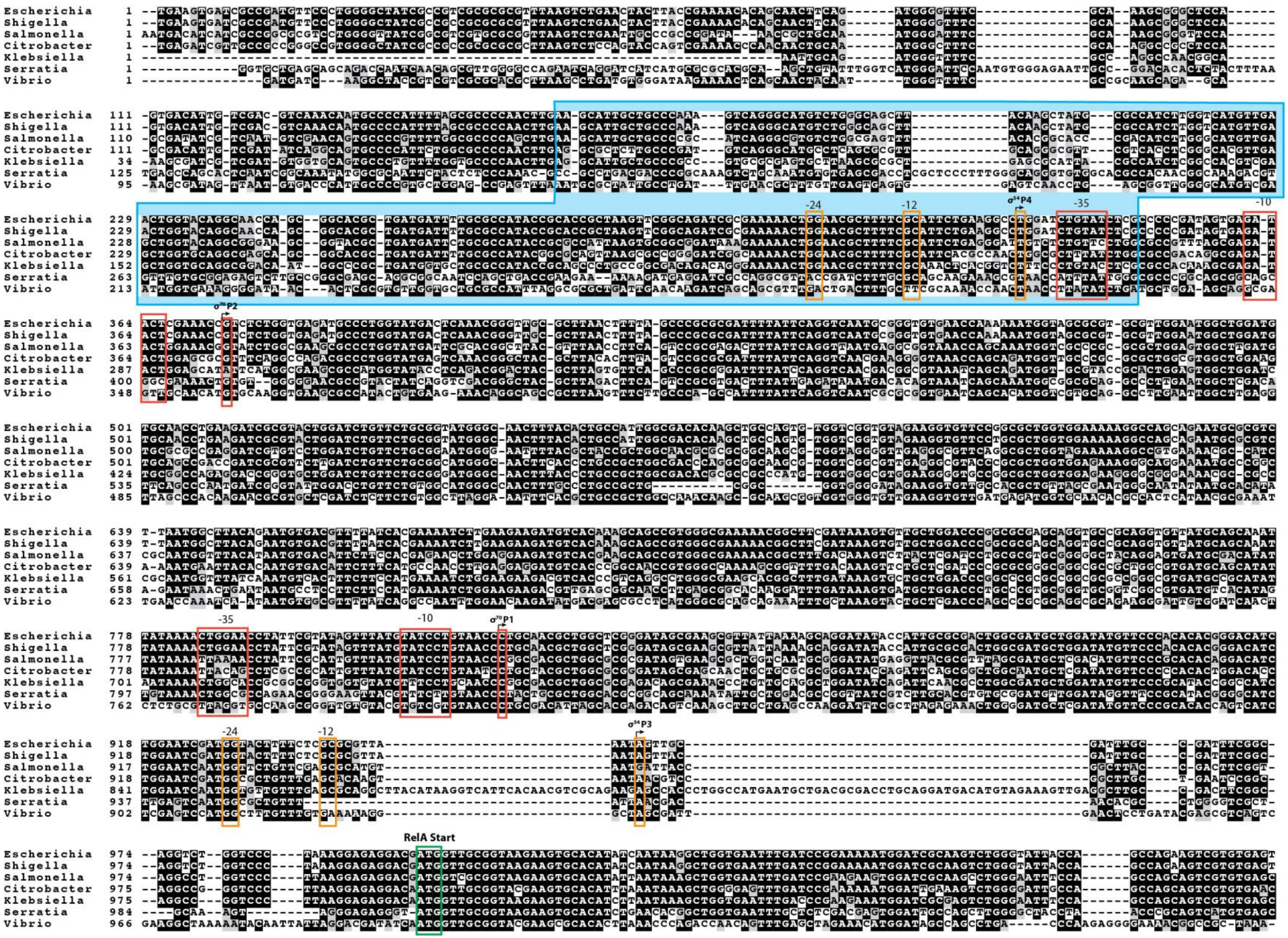
Escherichia
Shigella
Salmonella
Citrobacter
Klebsiella
Serratia
Vibrio
-24
-12
σ54 P4
-35
-10
σ70 P2
-35
-10
σ70 P1
-24
-12
σ54 P3
RelA Start

Supplementary Figure 5. The alignment of DNA sequences of *relA* regulatory regions (corresponding to positions –1,000 to +100 of RelA transcription start site of *E. coli*) from representatives of the Enterobacteriaceae family (*Shigella*, *Salmonella*, *Citrobacter*, *Klebsiella*, *Serratia* and *Vibrio*). The regions corresponding to the NtrC binding region and the promoters P1-4 are boxed in cyan (NtrC), red (P1 and P2) orange (P3 and P4) and the translation start site of RelA is indicated.

Transcription

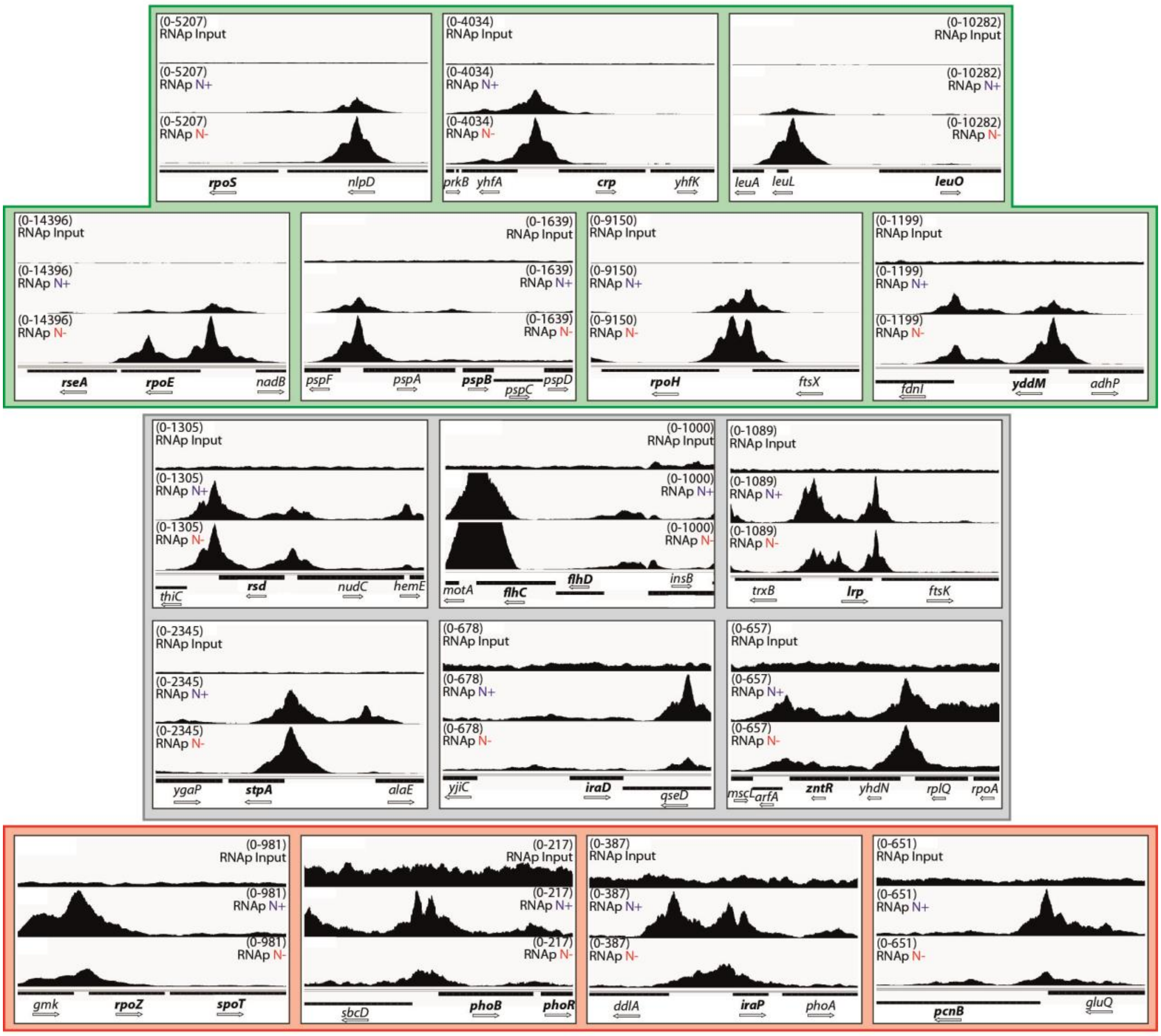

Stress adaptation

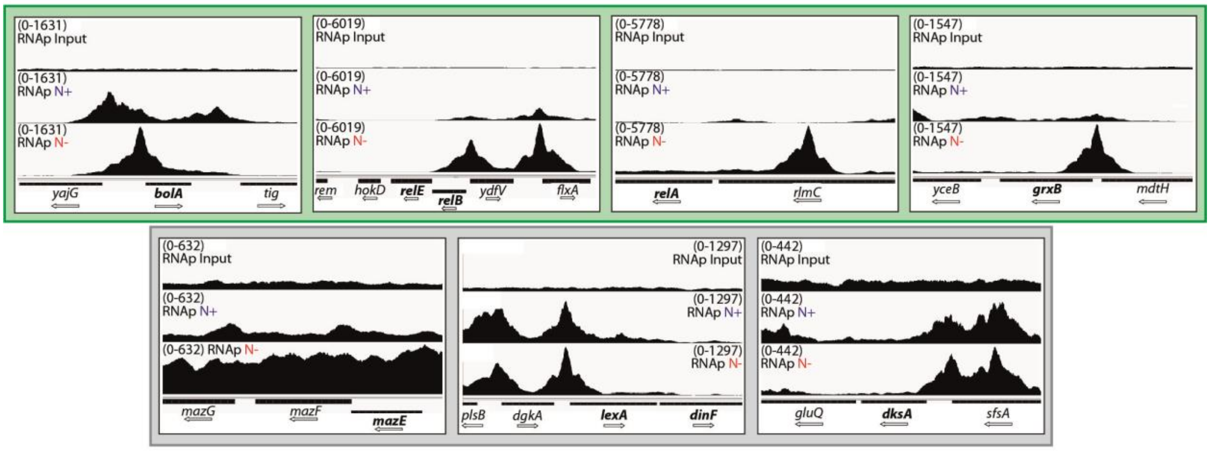

Translation

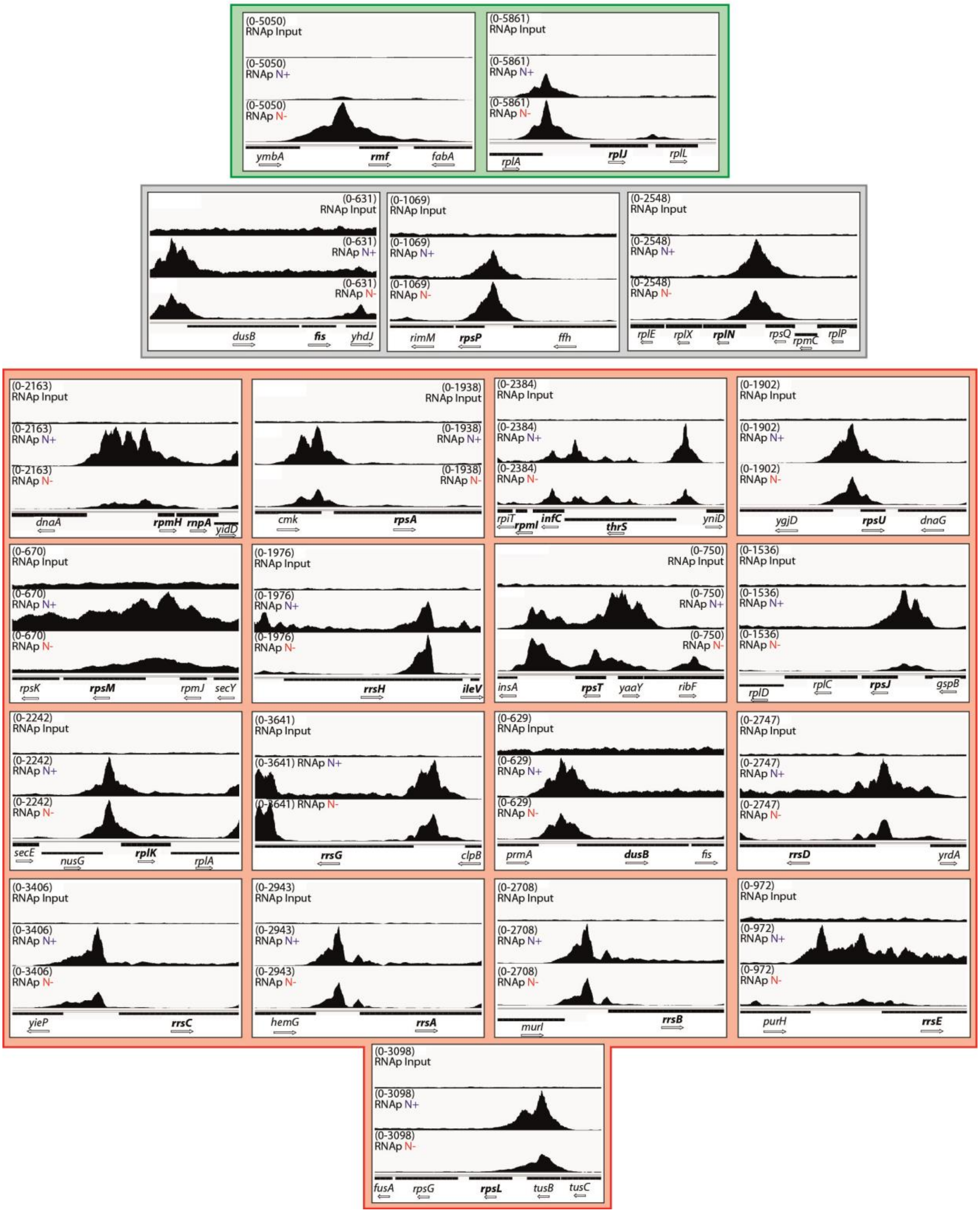

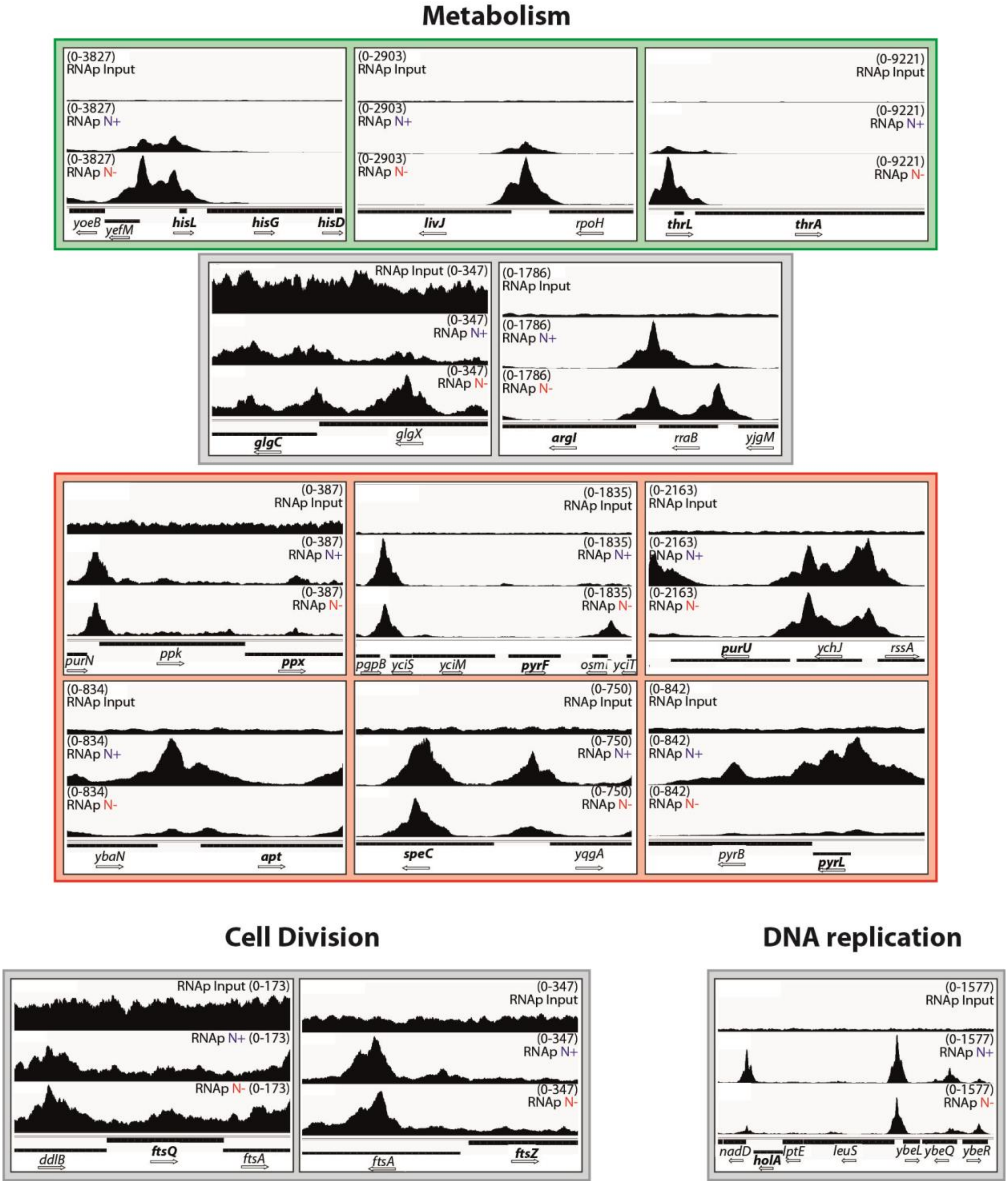

Supplementary Figure 6. Screenshots of Integrative Genome Viewer [1] with tracks showing the binding profiles (tag density) as measured by ChIP-seq of RNAp binding in N non-starved (denoted as N+) and N starved (denoted as N-) *E. coli* aligned against the upstream regions of all known ppGpp responsive promoters grouped into key cellular processes. A track with the input DNA control tag density (denoted as input) is shown for comparison. The screenshots in the green and red boxes denote promoters at which RNAp binding is positively

and negatively, respectively, affected by ppGpp during N starvation. The screenshots in gray boxes denote promoters at which RNAp binding remains unchanged at t=N+ and N-.

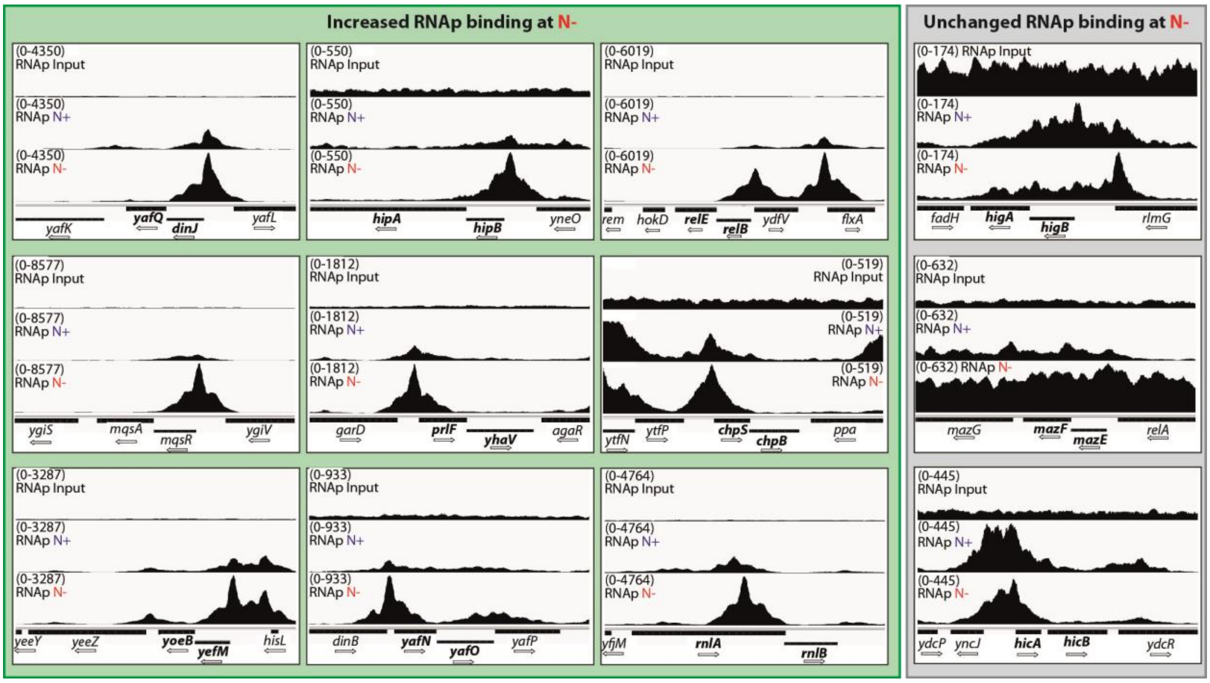

Supplementary Figure 7. Screenshots of Integrative Genome Viewer[1] with tracks showing the binding profiles (tag density) as measured by ChIP-seq of RNAp binding in N non-starved (denoted as N+) and N starved (denoted as N-) *E. coli* aligned against the upstream regions of toxin-antitoxin pair genes. A track with the input DNA control tag density (denoted as input) is shown for comparison. The screenshots in the green boxes denote promoters at which RNAp binding is positively affected by ppGpp during N starvation. The screenshots in gray boxes denote promoters at which RNAp binding remains unchanged at t=N+ and N-.

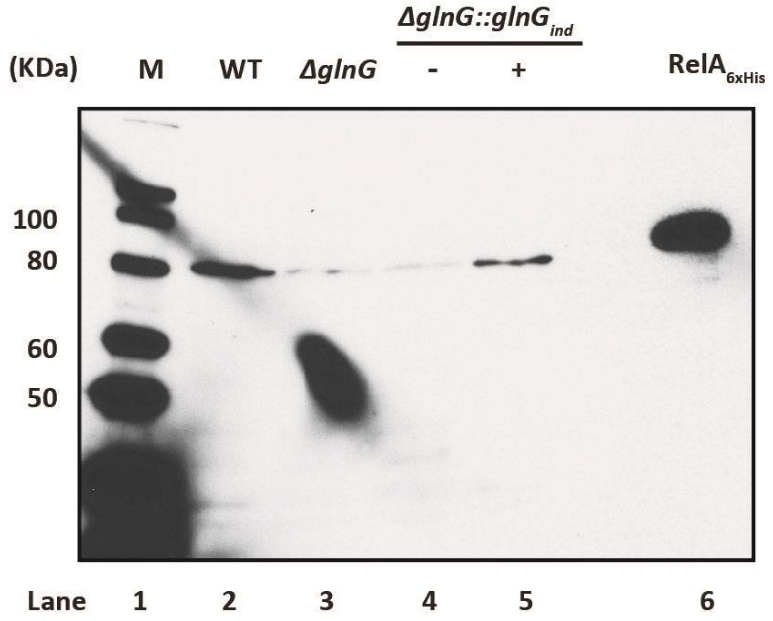

Supplementary Figure 8. Entire gel image of the RelA Western blot showing expression of RelA proteins in cells sampled at t=N- (as in Fig. 2C). Lane 1 contains the molecular weight marker and lane 6 contains purified *E. coli* RelA-6xHis protein.

Supplementary Table 1. Gene expression analysis of ppGpp-dependent promoters

Gene	Functional group	Increased ppGpp effect (predicted)[a]	RNAp ChIP binding N- vs N+	Fold change gene expression N- vs N+
rpoS	Transcription	+	+	↑ 4.59 ±0.75
crp	Transcription	+	+	↑ 2.41 ±0.36
leuO	Transcription	+	+	↓ 2.88 ±1.03
rpoE	Transcription	+	+	↑ 2.06 ±0.30
pspB	Transcription	+	+	↑ 5.88 ±1.05
rpoH	Transcription	+	+	↑ 7.23 ±1.04
yddM	Transcription	+	+	↑ 2.78 ±0.88
rsd	Transcription	+	nc	↑ 2.77 ±0.60
flhD	Transcription	-	nc	↓ 3.76 ±0.77
lrp	Transcription	+	nc	0.67 ±0.14
stpA	Transcription	-	nc	↑ 2.28 ±0.52
iraD	Transcription	+	nc	1.06 ±0.36
zntR	Transcription	+	nc	↑ 2.81 ±0.76
rpoZ	Transcription	-	-	0.76 ±0.21
phoB	Transcription	+	-	↑ 2.23 ±0.09
iraP	Transcription	+	-	↑ 3.23 ±1.29
pcnB	Transcription	-	-	0.64 ±0.06
bolA	Stress adaptation	+	+	↑ 5.69 1.80
relB	Stress adaptation	+	+	↑ 3.65 ±1.50
relA	Stress adaptation	+	+	↑ 6.78 ±0.75
mazE	Stress adaptation	-	nc	↑ 3.68 ±0.50
grxB	Stress adaptation	+	+	1.86 ±0.18
lexA	Stress adaptation	+	nc	↑ 6.01 ±0.58
dksA	Stress adaptation	-	nc	1.31 ±0.40
rmf	Translation	+	+	↑ 4.09 ±1.84
rplJ	Translation	-	+	↓ 4.33 ±1.30
fis	Translation	-	nc	↓ 18.83 ±3.52
rpsP	Translation	-	nc	↓ 8.69 ±1.51
rplN	Translation	-	nc	0.64 ±0.17
rpmH	Translation	-	-	↓ 8.01 ±1.78
rpsA	Translation	-	-	↓ 4.98 ±2.06
thrS	Translation	-	-	↑ 3.73 ±0.71
rpsU	Translation	-	-	↓ 21.70 ±3.36
rpsM	Translation	-	-	0.56 ±0.13
rrsH	Translation	-	-	No array data
rpsT	Translation	-	-	↓ 5.10 ±2.27
rpsJ	Translation	-	-	↓ 12.62 ±6.99
rplK	Translation	-	-	↓ 4.84 ±1.81
rrsG	Translation	-	-	No array data
dusB	Translation	-	-	↓ 26.35 ±4.17
rrsD	Translation	-	-	No array data
rrsC	Translation	-	-	No array data
rrsA	Translation	-	-	No array data

rrsB	Translation	-	-	No array data
rrsE	Translation	-	-	No array data
rpsL	Translation	-	-	↓ 5.77 ±1.64
hisG	Metabolism	+	+	↑ 2.63 ±0.45
livJ	Metabolism	+	+	↑ 3.92 ±1.71
thrA	Metabolism	+	+	↓ 4.33 ±2.4
glgC	Metabolism	+	**nc**	1.75 ±0.47
argI	Metabolism	+	**nc**	↑ 2.52 ±0.60
ppx	Metabolism	-	-	↓ 4.88 ±0.74
pyrF	Metabolism	-	-	↓ 6.41 ±1.72
purU	Metabolism	-	-	↓ 2.71 ±0.10
apt	Metabolism	-	-	↓ 56.18 ±5.37
speC	Metabolism	-	-	0.74 ±0.05
pyrL	Metabolism	-	-	0.64 ±0.10
ftsQ	Cell Division	+	**nc**	1.10 ±0.18
ftsZ	Cell Division	+	**nc**	↑ 4.16 ±1.44
holA	DNA replication	-	**nc**	↓ 3.04 ±0.56

Red indicates decrease, green indicates increase & grey indicates no change

[a] Predictions of changes in gene expression from an increase in cellular ppGpp levels are derived from Ecocyc[4]

Supplementary Table 2. TaqMan *E. coli* gene expression primer and probe sequences used in this study

Gene	Forward primer (5'-3')	Reverse Primer (5'-3')	FAM Reporter probe (5'-3')
glnK	GGGCATGCCGAGCTGTA	ATCAGCAATCGCCACATCAATTTT	CAGCGTCAATTTCCTG
relA	CGGGACATGAAGACCGGATT	ATCTCTTCCTGCCACGCAAT	CCTGGCTGCGTAAACT
16S RNA	CCCCCTGGACGAAGACTGA	GTGGACTACCAGGGTATCTAATCCT	TCCCCACGCTTTCG

Supplementary Table 3. EMSA probe and primer sequences

Probe name	Probe sequence 5’-3’ (Primer sequences bold and underlined)
glnA probe (344bp)	**GTCCCTTTGTGATCGCTTTCA**CGGAGCATAAAAAGGGTTATCCAAAGGTCATTGCACCAA<br>CATGGTGCTTAATGTTTCCATTGAAGCACTATATTGGTGCAACATTCACATCGTGGTGCA<br>GCCCTTTTGCACGATGGTGCGCATGATAACGCCTTTTAGGGGCAATTTAAAAGTTGGCAC<br>AGATTTCGCTTTATCTTTTTTACGGCGACACGGCCAAAATAATTGCAGATTTCGTTACCA<br>CGACGACCATGACCAATCCAGGAGAGTTAAAGTATGTCCGCTGAACACGTACTGACGATG<br>CTGAACGAGCACGAAGTGAAGTTT**GTTGATTTGCGCTTCACCGA**
relA probe (336 bp)	**CACAGCAACTTCAGATGGGG**TTTCGCAAAGCGGGCTCCAGTGACATTGTCGACGTCAAAC<br>AATGCCCCATTTTAGCGCCCCAACTTGAAGCATTGCTGCCCAAAGTCAGGGCATGTCTGG<br>GCAGCTTACAAGCTATGCGCCATCTTGGTCATGTTGAACTGGTACAGGCAACCAGCGGCA<br>CGCTGATGATTTTGCGCCATACCGCACCGCTAAGTTCGGCAGATCGCGAAAAACTGGAAC<br>GCTTTTCGCATTCTGAAGGCCTGGATCTGTATCTCGCCCCCGATAGTGAGATACTCGAAA<br>CCGTCTCTGGTGAGAT**GCCCTGGTATGACTCAAACG**
dicC probe (347 bp)	**ACGAATACCTGCTGCTTGTG**CAAGTTTTGTTTTTGAACCGAAATACAAAAGAGCGTCAGT<br>TTTAAGCATTTAAAACACCTTTATTGTTAGTCATAACTAACAAGATAGATGTTAACAAAA<br>ACATAGTCAATACGATTTAGCATTAGCTAACTATGGAAACAAAAAATTTAACTATCGGCG<br>AACGCATCAGGTATCGTCGGAAAAACCTCAAACACACCCAAAGGTCTCTTGCTAAAGCCC<br>TGAAAATCTCCCATGTGTCTGTATCACAATGGGAACGGGGTGATAGTGAACCTACAGGGA<br>AGAACCTTTTTGCCCTCAGTAAAGTAT**TGCAATGCTCACCAACATGG**
flgMN probe (312 bp)	**CTTGCTGCGCTTCGTTGAT**CAGCGCATCGGCAATTTTGCCGGTGTCCATTTTTAGTTCAC<br>CGTTACGAATCGCCAGTTTTAACGCTTCGACACGTTCAAGATTGATATCACTGCTGCCGG<br>GTTGCATCAGTTTTGCTTGCGCGTCGCTTAACGTCACACTGGTGCTGGTGGAGGCGGTTG<br>TTTTTGCCGCCCGGCTGTTCGTTACCGGCGCGTCAGTGGTTTCGCGCGGTTGAACGGTGC<br>TTACAGGCTTCAGAGGCGAAGTGCGATCAATACTCATGGTTTATTCCTCAT**TGAGGGCGC**<br>**TTTTATCATGTG**
ynfB probe (330 bp)	**ATCACTCTCAGCAAACGAATCG**GCCTGCTCGCTATTCTGCTGCCTTGCGCACTGGCATTG<br>AGCACAACTGTTCATGCCGAAACTAACAAACTGGTGATTGAGTCTGGCGACAGTGCACAA<br>AGCCGCCAGCACGCCGCTATGGAAAAAGAGCAATGGAATGACACGCGCAATCTGCGCCAG<br>AAAGTGAATAAACGCACTGAAAAAGAGTGGGATAAAGCCGACGCCGCTTTTGATAACCGC<br>GATAAATGTGAGCAAAGCGCCAACATCAATGCCTACTGGGAGCCCAATACTTTGCGCTGC<br>CTGGACCGTC**GAACTGGCCGCGTTATTACC**
fliC probe (359 bp)	**TTAGTACCGGTAGTGGCCTG**TACCGTCAGTTCACGCACACGCTGTAAGTTGTTGTTGATT<br>TCGGACAGCGCGCCTTCGGTGGTCTGCGCAACGGAGATACCGTCGTTGGCGTTACGGGCC<br>GCCTGAGTCAGGCCTTTAATGTTAGAGGTGAAACGGTTAGCAATCGCCTGACCCGCTGCG<br>TCATCCTTCGCGCTGTTAATACGCAAGCCAGAAGACAGACGCTCGATAGAACTCGACAGC<br>GCAGACTGGTTCTTGTTGATATTATTTTGAGTGATCAGCGAGAGGCTGTTGGTATTAATG<br>ACTTGTGCCATGATTCGTTATCCTATATTGCAAGTC**GTTGATTACGTATTGGGTTTCCA**
ssrS probe (346 bp)	**TTGAACAAGGTCGCATCACC**GAAAAAACTAACCAAAACTTTGAATGACACTTTTCGGTTT<br>ACTGTGGTAGAGTAACCGTGAAGACAAAATTTCTCTGAGATGTTCGCAAGCGGGCCAGTC<br>CCCTGAGCCGATATTTCATACCACAAGAATGTGGCGCTCCGCGGTTGGTGAGCATGCTCG<br>GTCCGTCCGAGAAGCCTTAAAACTGCGACGACACATTCACCTTGAACCAAGGGTTCAAGG<br>GTTACAGCCTGCGGCGGCATCTCGGAGATTCCCTTCTTATCTGGCACCAGCCATGACGCA<br>ACTACCAGAACTCCCACTGACATTAT**CCCGACAAGAAATCCGCAAA**
serA probe (331 bp)	**TCAGCCAGAATGCCCAATTG**CGTACCAATATGACCGTAGCCGATGATACCCAGCTTTTTG<br>CCGCGCGCTTCAAAAGAACCCGCCGCCAGTTTGTTCCACACGCCACGGTGCGCTTTAGCA<br>TTGGCTTCCGGCACGCCGCGCAATAGCAGCAGCAGTTCGCCAATCACCAGCTCCGCAACA<br>GAGCGCGTATTTGAGAACGGTGCGTTAAATACCGGGATCCCGCGCTTTGCCGCCGCATCC<br>AGATCAACCTGGTTTGTTCCGATACAGAAACAGCCAATAGCGACCAGTTTTTCTGCGGCG<br>TTGATCACGTC**TTCAGTCAGATGGGTACGGG**
yjcC probe (340 bp)	**CGGAACACATCATCGAGCTG**GCGCACAGCCTGGGGTTAAAAACGATCGCTGAAGGCGTCG<br>AAACTGAGGAGCAGGTTAACTGGCTGCGCAAACGCGGCGTGCGCTATTGCCAGGGATGGT<br>TCTTTGCGAAGGCGATGCCGCCGCAGGTGTTTATGCAATGGATGGAGCAATTACCCGCGC<br>GGGAGTTAACGCGCGGGCAATAAAATTACAGGCGGTGGCGATAATCGCTGGGAGTGCGAT<br>CAAACTGCCGACGGAAAACGCGGGAGAAGGTCTGCTGCGAGACATAACCCAGGTCCATTG<br>CGATATCAAAAATCGGACGCTC**GGTGGTGCGCAACTCAAC**

Supplementary Table 4. Primer sequences for 5'RACE PCR used in this study

Primer	Primer sequence (5'-3')
GSP1_*relA* (set A)	CGTGTCAATGTCCATACT
GSP2_*relA* (set A)	CGAGAGGATCTCCACCATCTCA
GSP3_*relA* (set A)	CATCTCAACACCACGCCAC
GSP1_*relA2* (set B)	CCAGCAACACTTTATCG
GSP2_*relA2* (set B)	GGCTGCTTTGTGACATCTTCTTC
GSP3_*relA2* (set B)	CTGTAAGCCATTAAGACGCGC

Supplementary References

1 Thorvaldsdóttir, H., Robinson, J. T. & Mesirov, J. P. Integrative Genomics Viewer (IGV): high-performance genomics data visualization and exploration. *Briefings in bioinformatics* **14**, 178-192, doi:10.1093/bib/bbs017 (2013).
2 Narlikar, L. & Jothi, R. ChIP-Seq data analysis: identification of protein-DNA binding sites with SISSRs peak-finder. *Methods in molecular biology (Clifton, N.J.)* **802**, 305-322, doi:10.1007/978-1-61779-400-1_20 (2012).
3 FinchTV (Geospiza Inc. http://www.geospiza.com/Products/finchtv.shtml).
4 Keseler, I. M. *et al.* EcoCyc: fusing model organism databases with systems biology. *Nucleic acids research* **41**, D605-612, doi:10.1093/nar/gks1027 (2013).
